# A Critical Evaluation of International Agreements Towards a Revised Categorization for Transfrontier Conservation Areas (TFCAs)

**DOI:** 10.1007/s00267-023-01872-3

**Published:** 2023-09-07

**Authors:** Francois Pieter Retief, Reece Cronje Alberts, Willem Daniel Lubbe, Claudine Roos, Dirk Petrus Cilliers

**Affiliations:** 1 Research Unit for Environmental Sciences and Management, North West University, Potchefstroom Campus, Potchefstroom, South Africa; 2 Protected Areas Research Group, North West University, Potchefstroom Campus, Potchefstroom, South Africa; 3 Faculty of Law, North West University, Potchefstroom Campus, Potchefstroom, South Africa

**Keywords:** Transfrontier Conservation Areas, Agreements, Evaluation, Categorization, Theory of Change (ToC)

## Abstract

Transfrontier Conservation Areas (TFCAs) are widely promoted as an international instrument to achieve certain conservation, cooperation and developmental goals, especially within the Southern African Development Community (SADC). In the SADC context, the status of TFCAs is categorized based on the extent to which international agreements have been signed. These agreements take different forms such as treaties, memorandums of understanding (MoUs), protocols and bilateral agreements. However, the efficacy of agreement-based approaches towards the categorization of TFCAs has been questioned because it does not acknowledge the implementation complexities of TFCAs and lacks a sound conceptual basis. This research evaluates the international TFCA agreements in SADC with a view to recommending a revised categorization. This is achieved by applying Theory of Change (ToC) to a sample of ten signed TFCAs agreements. The results show a lack of enforcement mechanisms, weak provision for implementation and poorly defined objectives. These weaknesses of agreement-based approaches can best be addressed by expanding the categorization of TFCAs to also include the extent of legislative and operational alignment. The revised categorization supports a more complete understanding of TFCA implementation.

## International Agreement-based Approach to TFCA Categorization

Internationally, the establishment of Transfrontier Conservation Areas (TFCAs) date back to the 1930s, with the establishment of the Waterton-Glacier International Peace Park between Canada and the USA (Quinn et al. 2012). The first TFCA in Africa was achieved in 1999, through a bilateral agreement between the governments of South Africa and Botswana to establish the Kgalagadi Transfrontier Park (Cumming et al. 2013). In 2013, the Southern African Development Community (SADC) Secretariat issued the SADC Programme for Transfrontier Conservation Areas with the mission, *“to develop SADC into a functional and integrated network of transfrontier conservation areas where shared natural resources are sustainably comanaged and conserved to foster economic and social development, tourism, and regional integration for the benefit of those living within and around TFCA and mankind at large”* (SADC 2013, p4). In the SADC context, TFCAs are defined as, *“ … areas or components of large ecological regions that straddle the boundaries of two or more countries, encompassing one or more protected areas, as well as multiple resource use areas”* (Ron 2007, p4). In its broadest sense, the purpose of TFCAs in SADC can be summarized as to promote conservation, cooperation and developmental goals in the region (Zunckel 2014; Vasilijević et al. 2015). Over the past two decades, TFCAs have become well established in the SADC region, with eighteen TFCAs covering terrestrial and marine environments at different phases of development (Blanken et al. 2022). Combined, these TFCAs cover more than one million square kilometres and include more than half of the protected area estate in southern Africa.

In the wake of international and regional support for TFCAs, considerable research has been done on different aspects, that include legal frameworks (Lubbe 2015; Mugadza 2019; Machaka 2021), TFCA performance evaluation (SADC 2020a, 2020b; Blanken et al. 2022), optimization of development potential (Weinert et al. 2020; Chitakira et al. 2022) and dealing with implementation challenges (Hanks 2003; Munthali 2007; Ron 2007; Muboku 2017; Malan 2015; Kachena and Spiegel 2019). Moreover, issues such as livestock and wildlife conflict (Thomson et al. 2013), implications of climate change (Zella et al. 2018), as well as tourism development (Zibanai 2019), received specific attention. However, there has been a scant reflection on the efficacy of the founding mechanism that determines the status and categorization of TFCAs in the first place, namely, international agreements.

Because TFCAs straddle national borders, legal formalization of these areas is based on international agreements that take different forms such as treaties, memorandums of understanding (MoUs), protocols and bilateral agreements. For the purpose of this research, we use the general term ‘international agreements’. In the SADC context, the status of TFCAs is categorized based on the extent to which international agreements have been signed. This current agreement-based categorization is as follows:

- *Category A—Established TFCAs:* These are TFCAs established through a treaty or any other form of legal agreement between the participating countries.
- *Category B—Emerging TFCAs:* These are TFCAs established on the basis of an MoU. The MoUs serve as instruments that facilitate negotiations of treaties to formally establish the respective TFCAs for eventual formalization to Category A.
- *Category C—Conceptual TFCAs:* These are TFCAs without an official mandate from the participating countries, but that have been proposed by SADC Member States as potential TFCAs.

The above categorization is important because it assigns a specific status to different TFCAs and implies a certain level of achievement in its formalization. There are various direct and indirect implications of such categorization, such as international/regional recognition, as well as funding opportunities and allocations. However, from a global perspective, there have been more than 250 000 international agreements signed that aim to foster cooperation (UN 2022) and valuations of these suggest an overall high level of ineffectiveness (Hathaway 2002; Cairncross 2004; Knapp and Franses 2009; Palmer et al. 2009; Hill 2010; Hafner-Burton et al. 2012; Iwata and Okada 2014; Koremenos 2016). For example, research by Hoffman et al. (2022) synthesized 224 primary evaluation studies and found that of the different types of treaties, environmental treaties were particularly ineffective. The main reasons for this are i) weak enforcement mechanisms, ii) poorly defined objectives, iii) a lack of resources and iv) commitment for/to implementation. There has also been much reflection on best practice for the development of international agreements (see for example: Gibson 2000; UN 2018; MFAT 2021). However, international agreements related to TFCA in SADC has not been evaluated. Therefore, their status in terms of weaknesses and/or best practice is unknown. Given that international agreements provide the basis for the categorization of TFCAs, a better understanding of their strengths and weaknesses could further inform categorization thinking. Therefore, this research aims to evaluate the international TFCA agreements in SADC with a view of recommending a revised categorization.

The next section explains the methodology, after which the results are discussed. The paper concludes with a conceptual framework towards a revised categorization of TFCAs in SADC.

## Evaluation Methodology

Theory of Change (ToC) is a widely applied performance monitoring and evaluation method, especially in relation to policy implementation mechanisms (Mason and Barnes 2007; Rogers 2008; Brouselle and Champagne 2011; Van der Sluijs 2017; McConnell 2019). Moreover, international development agencies apply ToC as a best practice evaluation method for their programmes (Weiss 1995; Davidson 2004; Stein and Valters 2012; USAID 2015; Allen et al. 2017). Many countries in the SADC region uses ToC as their preferred method to evaluate the performance of policy instruments (Rossouw and Wiseman 2004; Alberts et al. 2020; Moolman et al. 2022). Recently, ToC has been applied specifically within the southern African conservation context to evaluate the performance of protected area systems, as well as different types of protected areas (Alberts et al. 2022; Retief et al. 2022). Therefore, the application of ToC to the evaluation of TFCA agreements is well justified within the SADC context.

There are two distinct approaches to the application of ToC (Allen et al. 2017; Retief et al. 2022). From a methodological design perspective, it is important to distinguish between these two approaches. The first approach is to use ToC to design a new intervention or legal mechanism to achieve certain policy outcomes. The second approach is to evaluate an existing intervention or legal mechanism, by identifying certain key assumptions and risks to the intervention delivering intended outcomes and/or functioning as intended. In case of the latter, the intervention or legal mechanism has already been designed and, therefore, the evaluation deals with ‘existing design’ and not ‘preferred design’ (as is the case with the first approach). In the case of TFCA agreements, we followed the second approach as international agreements (in their various forms) are already identified and mandated as the preferred TFCA implementation mechanism.

### Applying ToC to TFCA International Agreements

**Fig. 1:**
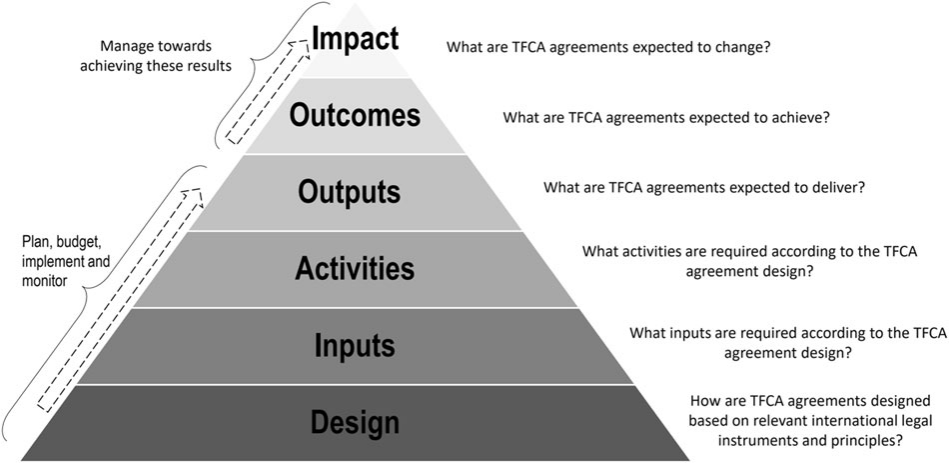
Results-based pyramid for evaluation of TFCA agreements

The ToC method produces a causal understanding of the existing preferred legal mechanism design (in this case international agreements), presented in the form of a so-called ‘causal narrative’ and related ‘ToC map’. The causal narrative and ToC map are developed against a sequence of different evaluation components namely: design, inputs, activities, outputs, outcomes, and impacts. These components are conceptualised and illustrated in the form of a pyramid, namely the ‘results-based pyramid’. For the purpose of this research, we adapted the pyramid specifically for the evaluation of international agreements in the context of TFCAs as illustrated in Fig. 1.

The ToC evaluation requires objectively answering the following generic evaluation questions, namely:

- *Design component:* How are TFCA agreements designed based on relevant international legal instruments and principles?
- *Input component:* What inputs are required according to the TFCA agreement design, to achieve prescribed outputs, outcomes and impacts?
- *Activities component:* What activities are required according to the TFCA agreement design, to achieve the prescribed outputs, outcomes and impacts?
- *Output component:* What are TFCA agreements expected to deliver?
- *Outcome component:* What are TFCA agreements expected to achieve?
- *Impact component*: What are TFCA agreements expected to change?

The main methodological actions are to i) articulate from the design component an accurate causal narrative explaining the mechanism design, ii) illustrate the causal narrative on a ToC map, iii) identify key assumptions underpinning the causal narrative and iv) translate these key assumptions into evaluation criteria.

The ToC causal narrative, map and assumptions were developed through a specialist workshop. The specialists all have PhDs qualifications with more than 15 years’ experience in the following fields of expertise: conservation planning, environmental law, environmental management and environmental science. It must be stressed that in line with the second ToC approach, the aim of the specialist workshop was to develop the causal narrative and ToC map based on the current TFCA agreement design, and not on a preferred design (i.e. if the agreements were to be designed from scratch).

### Causal Narrative and ToC Map

**Fig. 2:**
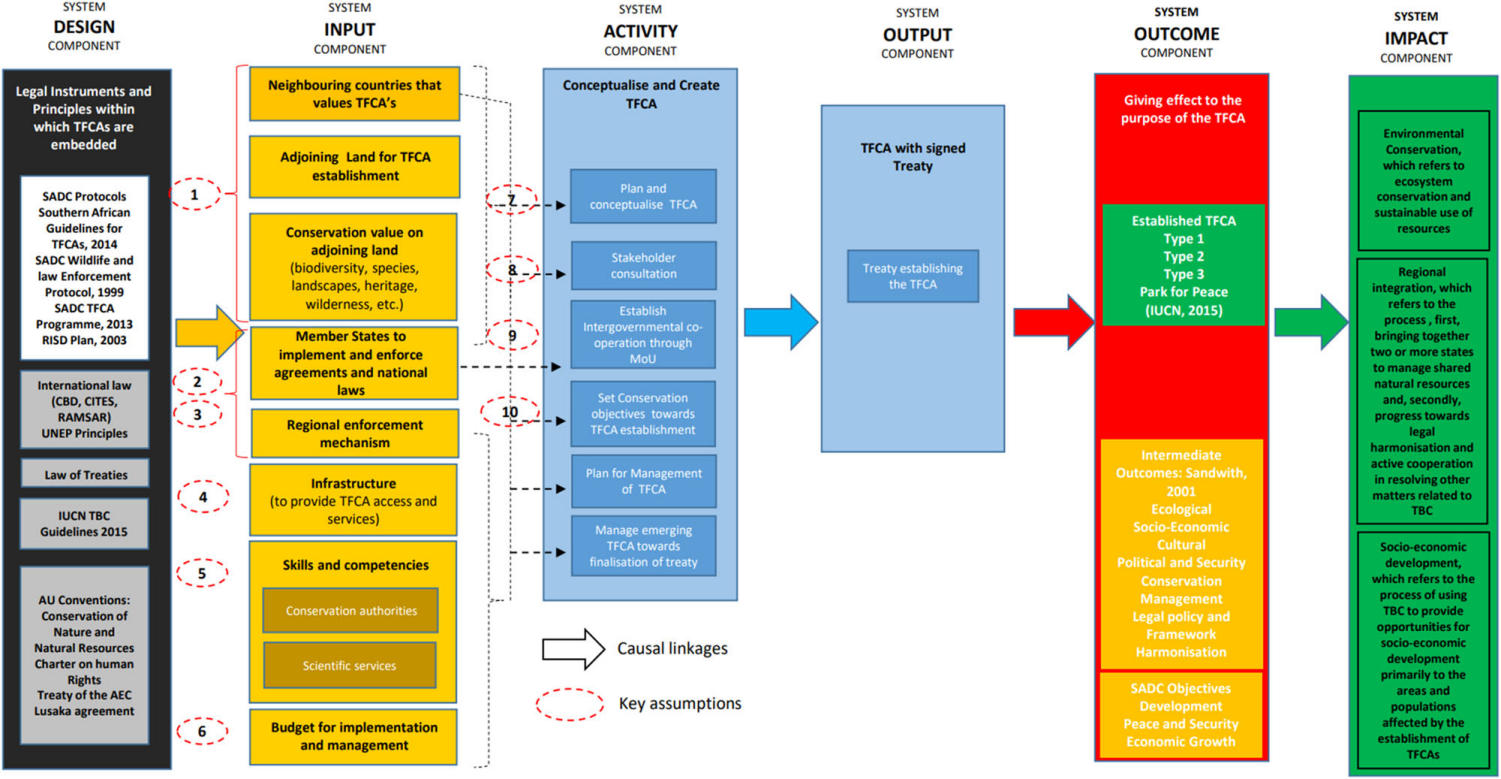
ToC map for TFCA international agreements in SADC

This section explains the causal narrative against the different ToC components and how it relates to the ToC map (Fig. 2). The key assumptions for evaluation are derived from this narrative and indicated on the ToC map, numbered 1 to 10 on the map. Table 1 shows how these assumptions relate to and are translated into ten key evaluation criteria.

- *Design component*: The design component deals with the legal instruments and embedded legal principles relevant to TFCA agreements. These range from international to regional policy and legislation originating from the UN, the African Union and SADC. Moreover, evaluating international agreements against the above aims and goals of TFCAs requires benchmarking the agreements against best practice legal principles for TFCAs. These principles are distilled from, among others, the International Law Association New Delhi Declaration of Principles of International Law Relating to Sustainable Development (2002 A/Conf/199/8) and the UNEP ‘Principles of Conduct in the Field of Environment for the Guidance of States’ in the ‘Conservation and Harmonious Utilization of Natural Resources shared by two or more States’ report, 1978 (UNEP/IG -7/3). It is important to note that the content of the documents has become increasingly relevant in international judicial proceedings and, therefore, in the design of international agreements. This is especially true in the context of the African Court on Human and Peoples’ Rights (ACHPR) and the African Commission on Human and Peoples’ Rights. Therefore, the design component of the ToC relies on international law and policy to provide the basis for the international TFCA agreements.
- *Inputs component:* The input component deals with the resources that are required for the delivery of the activities and the output components (Weiss 1995; Connell and Kubisch 1998; DPME 2011; Thornton et al. 2017). The process of identifying the key inputs for the SADC TFCA system resulted in intense deliberation during the development of the causal narrative, due to the myriad of possible inputs ranging from specific operational factors (such as day-to-day TFRCA management) to larger strategic and systemic inputs (for example the establishment of multi-member state TFCAs), coupled with the fact that the inputs are not explicitly provided for in the legal and policy framework making up the design component (unlike for example the outcome and impact components). Therefore, this section shares what were considered key inputs, summarised in the second column of Fig. 2. TFCAs require firstly neighbouring countries that value conservation and TFCAs as an instrument. The rationale is that the entire system is based on cooperation and agreement. In theory, should adjoining member states not value conservation and TFCAs, then the concept is moot within that particular context. Adjoining conservation-worthy areas or marine areas for inclusion in the TFCA is a further input, which forms the building block for the TFCA concept. For TFCAs to function there is a requirement for member states to implement and enforce the protected area and conservation laws which have been passed. Relevant infrastructure is required to allow for the management and operation of TFCAs in line with relevant objectives. Arguably, one of the most important inputs is that relating to skills and competencies, because for TFCAs to function properly, sound leadership, management, and scientific skills are required, coupled with competent management authorities. Lastly, the above inputs are strongly reliant on the requisite financial budgets and resources to implement the TFCA.
- *Activity component:* The ToC approach determines that the activity component deals with the process or actions that use the inputs (described in the previous section) to produce the desired output and ultimately the desired outcomes (Weiss 1995; Connell and Kubisch 1998; DPME 2011; Thornton et al. 2017). The identified activities relevant to TFCA establishment are identified in column three of Fig. 2. These are: the planning and conceptualization of the relevant TFCA; stakeholder consultation, which is a pre-requisite for TFCA establishment, as well as relevant intergovernmental cooperation, which is in theory supported by the signing of an agreement (such as MoU) by the relevant member states. Furthermore, relevant conservation goals and objectives must be identified and established for the TFCA. The management of the TFCA by member states must be planned and formalised. The emerging TFCA must then be managed towards the finalisation of the TFCA treaty and become what is considered an established TFCA.
- *Output component:* The output component represents the outputs culminating from the design, inputs and activities components. In the case of SADC TFCAs, the actual output is an established TFCA, that comply with the three categorizations and overall definition provided in the first section above.
- *Outcome component:* The outcome component entails that which should be achieved by the particular output. In the case of SADC TFCAs, the outcome component would be represented by an established TFCA which falls within either type 1, 2, or 3 designations as identified by the IUCN (Erg et al. 2015; SADC 2020b). Type 1 refers to transboundary protected areas that are ecologically connected across one or more international boundaries and involves some form of cooperation. This would entail formally declared protected areas within member states which are ecologically connected across the relevant boundaries. Type 2 refers to transboundary conservation landscapes or seascapes, which may include protected areas. Finally, Type 3 refers to transboundary migration conservation areas comprising wildlife habitats. Certain intermediate outcomes are identified for TFCAs by Sandwith et al. (2001) as depicted in Fig. 2. Furthermore, SADC has recognised that intermediate outcomes relating to TFCAs relate to development, peace and security, and economic growth in the region.
- *Impact component:* The impact component represents the results of achieving certain outcomes. Within the TFCA context the impact component would be the achievement of the SADC TFCA objectives, namely: environmental conservation, which refers to ecosystem conservation and sustainable use of resources. Secondly, regional integration which refers to the process, first, bringing together two or more states to manage shared natural resources and, secondly, progress towards legal harmonisation and active cooperation in resolving other matters related to transboundary conservation. Lastly, socio-economic development that provides opportunity for socio-economic development in the establishment of TFCA regions (SADC 2020b).

**Table 1:** Key assumptions and criteria for the evaluation of TFCA agreements

Nr	Key Assumptions (*see ToC map*—Fig. 2)	Key Evaluation Criteria
1	Land included in the agreement is valued by parties as having conservation value.	To what extent is the land included in the agreement valued by parties as having conservation value?
2	Provision is made in the agreement for a functional regional enforcement mechanism.	To what extent is provision made in the agreement for a functional regional enforcement mechanism?
3	Provision is made in the agreement for functional national conservation enforcement mechanisms.	To what extent is provision made in the agreement for functional national conservation enforcement mechanisms?
4	The agreement makes provision for infrastructure to be provided.	To what extent does the agreement make provision for infrastructure to be provided?
5	The agreement makes provision for conservation skills and competencies to be provided.	To what extent does the agreement make provision for conservation skills and competencies to be provided?
6	The agreement makes provision for budget to be provided for implementation and management.	To what extent does the agreement make provision for budget to be provided for implementation and management?
7	The boundaries of the area are defined in the agreement.	To what extent is the boundaries of the area defined in the agreement?
8	The agreement is supported by stakeholder consultation.	To what extent is the agreement supported by stakeholder consultation?
9	Intergovernmental cooperation is formalized in the agreement.	To what extent is intergovernmental cooperation formalized in the agreement?
10	Conservation objectives are clearly defined in the agreement.	To what extent is conservation objectives clearly defined in the agreement?

### Evaluation Criteria and Process

The previous section described the causal narrative against the ToC map—as shown in Fig. 2. The causal narrative produced ten key assumptions underpinning the design and intended functioning of the agreements. These assumptions are indicated and numbered on the ToC map from 1 to 10. Table 1 lists the ten assumptions translated into key evaluation criteria.

The criteria (Table 1) are applied to the content of the different international agreements and evaluated according to the following scores:

- *‘A’ score*—Conformance to the criterion: Relevant provisions included with no significant omissions.
- *‘B’ score*—Partial conformance to the criterion: Relevant provisions partially included with some level of omission evident.
- *‘C’ score*—Non-Conformance to the criterion: Relevant provisions not included with significant omissions.

‘Letters’ rather than ‘numbers’ are used as evaluation symbols to discourage crude aggregation. The evaluation should not only record the chosen assessment symbols, but also record, in a brief summary, the principal justification for the evaluation score. This serves to avoid ‘over-mechanical’ evaluations and support transparency and validity of results.

The five specialists evaluated the content of each of the international agreements after which individual scores were discussed and a consensus score was agreed. The combined discussion centred around the individual justification of different scores. It is important that the agreements be evaluated independently by multiple reviewers and that any differences in the results be systematically examined and resolved. Ultimately, the evaluation results produced a high level of agreement between reviewers on the strengths and weaknesses of the different agreements. Strengths in relation to the evaluation would suggest areas where the agreements function as designed/intended, while weaknesses would suggest areas where the agreements do not function as designed. The advantage of specialist reviews is that it produces (and assumes) a high level of competence and understanding by those doing the review. The resultant consensus outcome implies a high level of assurance based on specialist expertise.

### Sample of TFCA Agreements Evaluated

In total, a sample of ten TFCAs, for which international agreements have been signed, were included in the evaluation. The international agreements related to these TFCAs take different forms with four treaties, three agreements, two MoUs and a protocol.

**Fig. 3:**
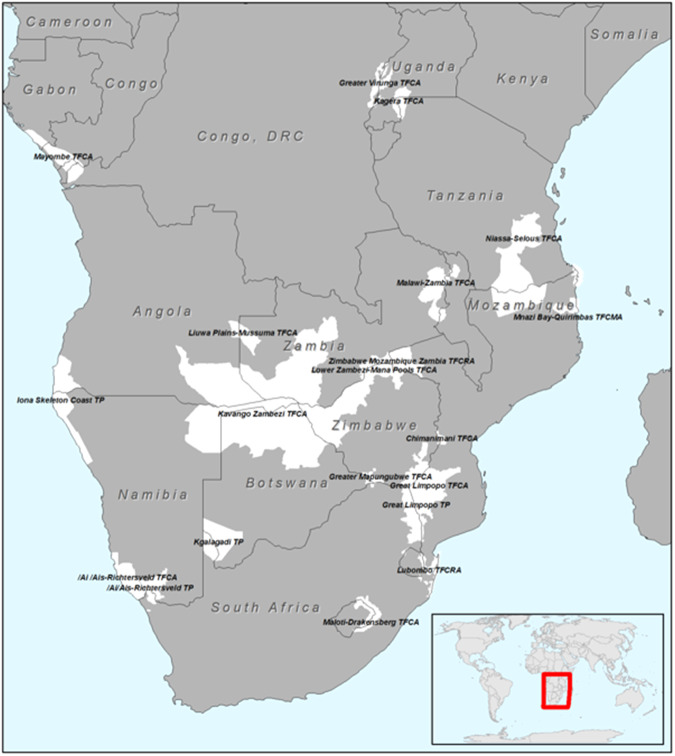
Location of different TFCAs in the SADC region

Figure 3 provides the location of these ten TFCAs. However, due to potential political and partner country sensitivities related to the evaluation of individual TFCA agreements, results are kept anonymous and not discussed in relation to any specific TFCA. Rather, patterns in results across the ten cases are discussed.

## Data Analysis and Discussion

Table 2 provides the results of the evaluation, where the ten TFCA cases (Fig. 3) were evaluated against the criteria provided in Table 1. The following sections discuss the strengths and weaknesses of the international agreements against the ten evaluation criteria. These strengths and weaknesses relate to the international agreement design as explained in the causal narrative and ToC map. The first general point to highlight is that no correlation could be found between different forms of international agreements (i.e. treaties, agreements, MoUs and protocols) and overall TFCA performance. It, therefore, seems to matter little what form the agreement takes, or what it is called. The second general point is that the term ‘TFCA’ is only used in three of the treaties evaluated. The majority (four) refer to ‘Transfrontier Parks’ (TFPs) and the remainder respectively to ‘TFC and Resources Area’, ‘Transfrontier Development and Conservation Area’ and ‘Agreement on the Conservation and Management of an Ecosystem’. The format and terminology used across the agreements, therefore, differ widely, in contradiction to the categorization that explicitly refers to only TFCAs, treaties and MoUs.

**Table 2:** International agreement evaluation results across TFCAs

Evaluation criteria (See ToC map—Fig. 2)	TFCA Agreements									
	1 Treaty	2 MoU	3 Agreement	4 Treaty	5 Treaty	6 Treaty	7 Protocol	8 MoU	9 Agreement	10 Agreement
1. To what extent is the land included in the agreement valued by parties as having conservation value?	B	A	A	C	B	B	C	B	B	A
2. To what extent is provision made in the agreement for a functional regional enforcement mechanism?	C	C	C	C	C	C	C	C	C	C
3. To what extent is provision made in the agreement for functional national conservation enforcement mechanisms?	B	A	A	C	B	C	C	B	C	B
4. To what extent does the agreement make provision for infrastructure to be provided?	B	C	A	C	C	C	C	C	C	C
5. To what extent does the agreement make provision for conservation skills and competencies to be provided?	C	B	A	C	C	C	C	C	C	C
6. To what extent does the agreement make provision for budget to be provided for implementation and management?	B	B	A	B	C	B	B	B	B	C
7. To what extent is the boundaries of the area defined in the agreement?	A	A	A	A	B	A	C	B	B	B
8. To what extent is the agreement supported by stakeholder consultation?	B	C	C	C	C	C	B	B	C	C
9. To what extent is intergovernmental cooperation formalized in the agreement?	A	A	A	B	A	A	B	A	A	A
10. To what extent is conservation objectives clearly defined in the agreement?	A	B	A	C	B	B	B	B	B	B

Evaluation Scores: A—Conformance; B—Partial conformance; C—Non-Conformance

### Strengths

The results show the best scoring criterion to be Criterion 9, which reflects on the extent to which intergovernmental cooperation is formalized in the agreement. Eight of the ten agreements scored ‘A’ and two scored ‘B’. The reason for the scoring is that all agreements made explicit provisions for ministerial committees with related powers and functions. The institutional arrangements and powers and functions vary between two and six-tiered systems. Although the agreements do provide explicit and detailed arrangements for formalised cooperation, that suggest compliance with Criterion 9, they seem to prefer complicated, multi-tiered and, ultimately, high-level centralized decision-making. Coordination and implementation functions end up relying on ministerial sign-off. Moreover, at the implementation level, there seems to be a lack of coordination arrangements. Ideally, implementation needs to be coordinated and decision-making devolved to coordination structures. The current arrangements mostly require high-level, centralized decision-making with implementation vested in member states, which produce cumbersome uncoordinated decision-making. Few of the agreements formalized a secretariat function with a specific location within one of the member states. The decision-making authority of the secretariat function is also scantly addressed, as a critical component of an effective institutional system. Therefore, although this aspect is well addressed, the practical implications may present serious challenges during implementation.

Another strength seems to be Criterion 7, which deals with the extent to which TFCA boundaries are defined in the agreements, which scored five ‘A’s, four ‘B’s and a ‘C’. Clearly much thought and planning have gone into the spatial extent of the proposed TFCAs, even if still at a very strategic and high-level scale. The majority of agreements include maps, and in some instances, detailed coordinates. Boundary descriptions generally seem clear when referring to formally protected areas that are included in the TFCA, while the buffer or corridor areas are less clearly described. The agreements do make general provision for options and mechanisms to expand the TFCA, but the procedures to affect such expansion is not always clear. However, having clear boundaries is a positive feature of the agreements that greatly support decision-making, planning and implementation.

The defined purpose of TFCAs is to promote conservation within a mixed land-use context. The conservation value of certain land included in the agreement is well recognized as having conservation value through formal protection status. In this regard, Criterion 1 scored three ‘A’s, five ‘B’s and two ‘C’s. The understanding in relation to this criterion is that the more formally protected areas included in the agreement the higher the acknowledged conservation value of the land. The combination of protected areas and other land uses implies that the conservation value of certain areas included in the TFCA is uncertain, otherwise, they would per definition be formally protected. The TFCA agreements, therefore, include a meaningful portion of land recognized as having conservation value.

### Weaknesses

The data analysis produced the following key weaknesses: i) the lack of enforcement mechanisms (see Criterion 2, 3 and 8); ii) weak provision of resources for implementation (Criterion 4, 5 and 6) and iii) the lack of clear objectives (Criterion 10).

Criterion 2 performed the weakest with all cases scoring ‘C’. This means that no meaningful provision is made for regional enforcement mechanisms. The lack of regional enforcement mechanisms is not unique to TFCAs in SADC, but is an international feature of environmental treaties, as recently shown by Hoffman et al. (2022). The TFCA agreements vary in terms of dispute resolution and mediation provisions, from vague calls to be settled ‘amicably’ between countries themselves, to more specific third-party mediation by the SADC secretariat, African Union (AU) or the International Court in Hague. The strong voluntary nature of the agreements means that they rely almost exclusively on an incentive basis or a win-win scenario for the parties involved, with no discernible consequences for parties who negate responsibilities. The only real recourse to deal with grievances or disputes seems to be withdrawal from the agreement itself. Criterion 3, dealing with the provision of national-level enforcement mechanisms scored slightly better, with two ‘A’s, four ‘B’s and four ‘C’s. This is because national-level enforcement already exists, linked to the enforcement mandate in local conservation and protected areas legislation. However, it is unclear what the national enforcement options would be outside formally protected areas. These areas would be subject to general national policy and legislation on land use control and/or ownership. In view of the inherent weaknesses of environmental agreements in terms of regional enforcement, the most viable option forward would be to recognize opportunities for better alignment and strengthening of national policy and legislation of partner countries. Enforcement is, therefore, not vested in the international agreements but rather at the national level.

Provision for stakeholder engagement is particularly weak, with Criterion 8 scoring seven ‘C’s and three ‘B’s. Very limited provision is made for participation and or stakeholder engagement within the institutional structures described in the agreements. The weaknesses of highly centralized decision-making structure of TFCAs have been highlighted with a call for the voices of local stakeholders, as the *de facto* resources managers, to be more prominent (Cumming et al. 2013). In practice, Malan (2015) also found very limited information sharing among TFCA stakeholders and that conflict is only dealt with at a very late stage, mostly driven by emergencies or crises such as fires or poaching of rare species. Moreover, a need has been identified in practice to allow individuals affected by the operational rules of the TFCA to participate in modifying these rules. The opacity of the current decision-making system described in the agreements does not allow for such participation. Establishing monitoring procedures would also potentially increase accountability by stakeholders and resource users and graduated sanctions against violators of the TFCAs’ operational rules.

Criterion 4 and 5 scored particularly poorly with 16 ‘C’s, 2 ‘B’s and 2 ‘A’ between them, which means that the agreements failed to make provision for infrastructure provision and skills. The agreements merely make general reference to the need for sharing of skills, without provision for mechanisms to create or source skills where none exists. Research has suggested that the paucity of dedicated staff from different signatories is a key failure of TFCAs (Malan 2015). Practical suggestions have been for each TFCA to set up a permanent, physical institution or entity dedicated to infrastructure and skills development. The secretariats referred to in the agreements are insufficient, because they are assigned primarily an administrative function, with no reference to skills and/or infrastructure development.

Financial provisions (Criterion 6) are dealt with slightly better with seven ‘B’s, two ‘C’s and an ‘A’. However, financial provisions are dealt with mainly at an institutional level and not at a commitment level. Therefore, financial institutions are identified but only one case made explicit provision for contributions and sharing of funds. Some cases did include a reference to ‘equal allocation’, but it was difficult to understand and ascertain what the reference/s meant exactly. Institutionally, the majority of agreements allocated budgeting and financial arrangements, as well as oversight at the individual state or national level while three cases set up what is referred to as ‘TFCA Funds’. These provisions are supposed to facilitate the sourcing and administration of funds in coordination with the SADC secretariats and party signatories. Ultimately, the funding of TFCAs rely on budget allocations from signatories (mostly existing protected area budgets) and international funding streams. The latter has been a significant contributor to TFCA implementation over the past two decades. It is fair to say that significant funding shortages remain a key challenge for TFCAs and, therefore, agreements seem to fall short of addressing this issue.

Criterion 10 evaluated to what extent the agreements set clear conservation objectives. The results show a mostly average to poor result with seven ‘B’s, two ‘A’s and a ‘C’. All but one agreement made specific reference to conservation objectives, but they are very broad and poorly defined, conflicting in some cases and idealistic. Agreements require clearly defined objectives to provide direction for implementation and benchmarks against which to monitor. We are of the opinion that the current objectives do not serve to inform management and or implementation plans and provide very limited direction to signatories in terms of direction. It is likely that the successes of TFCAs currently experienced have very little to do with the signed agreements, but more to do with the effective alignment of operation and management plans between signatories at an operational level. We suspect that these operation and management plans have no relation to the objectives contained in the agreements, but this suspicion/ assumption will have to be tested.

### Revised Categorization of TFCAs in the SADC Region

**Fig. 4:**
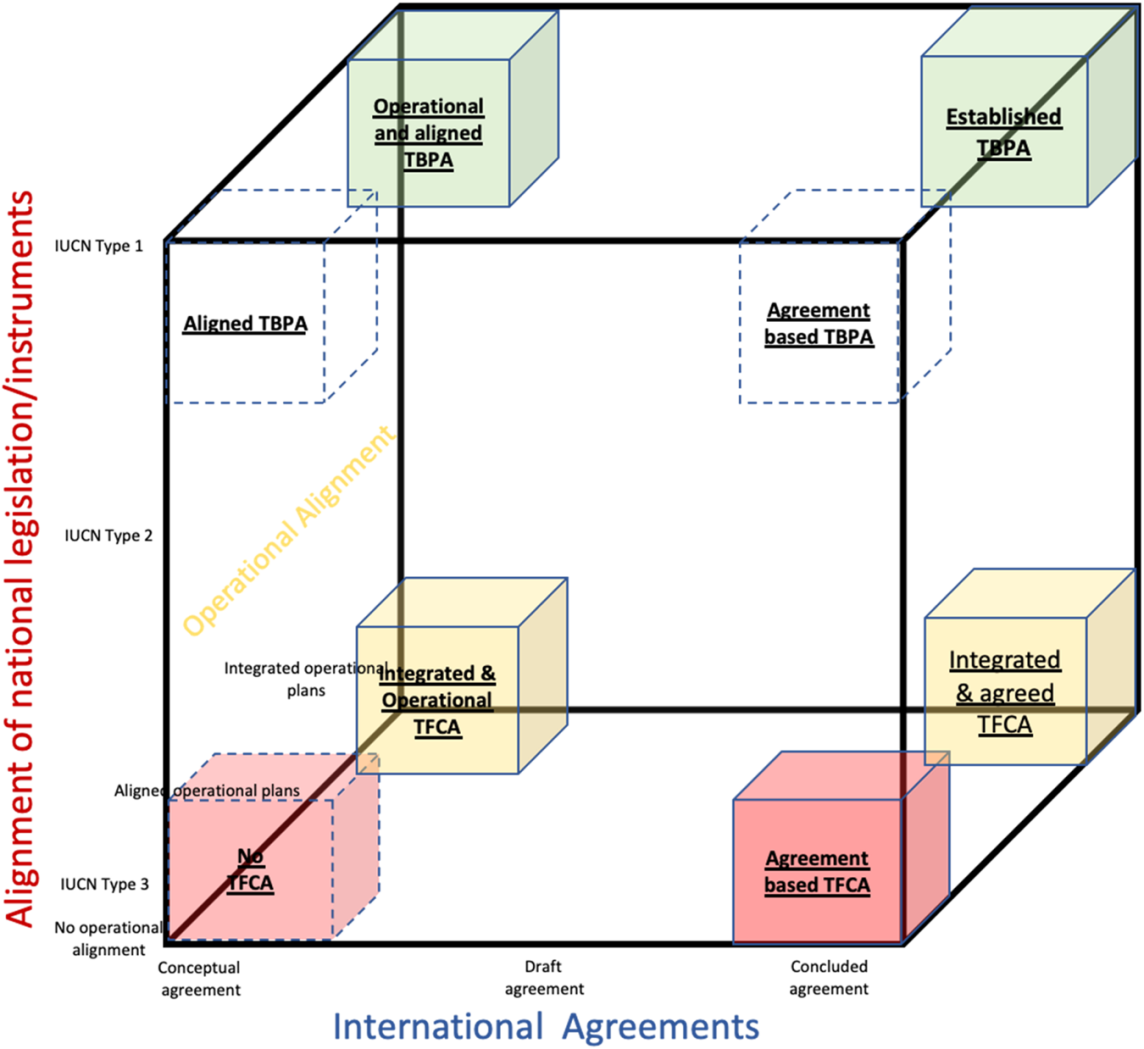
Example of revised categorization for TFCAs in SADC

In keeping with the aim of this paper, we provide a recommendation towards revising the current SADC TFCA categorization, based on the weaknesses of the evaluated international agreements. With a view to address the weaknesses, we suggest that agreement-based categorization be expanded beyond the recognition of signed agreements to also include two additional categorizations, namely: (i) the extent to which national-level conservation policy and legislation have been aligned; and (ii) the extent to which management and operational arrangements have been integrated. These three categorizations provide a more comprehensive and holistic perspective on the implementation status of TFCAs. Figure 4 represents these three categorizations representing three axes (x, y and z) within a three-dimensional cube. The three axes represent the following:

- *Status of international agreements:* The x-axis is a spectrum of agreements underpinning TFCA conceptualization and establishment. This axis progresses from conceptual agreement to draft agreement, culminating in a final (concluded) agreement between member states supporting and establishing the TFCA. TFCAs may, thus, be placed along this spectrum according to the agreements applicable to each. For example, TFCAs without a formal agreement would be aligned towards the left of the x-axis, whilst those with formalized and signed agreements would be placed on the right.
- *Extent of international policy and legislative alignment:* TFCA alignment could also be in relation to the extent of national level policy and legislative alignment (y-axis). To standardize the IUCN typology (type 1 = transboundary protected area, type 2 = transboundary conservation landscape and/or seascape, and type 3 = transboundary migration conservation areas), relevant to TFCAs could be used as a reference for national typologies. Ultimately, national level policy and legislation needs to be aligned, because this would potentially address the weak enforcement potential of international agreements highlighted above.
- *Extent of management and operational alignment:* TFCAs could also be aligned with the z-axis, which denotes the level of operational alignment between member states and the relevant conservation agencies. TFCAs may be placed along the z-axis denoting either no operational alignment, operational alignment or integrated operational alignment, which culminates in a joint and integrated operational management plan for the TFCA.

Placing a specific TFCA within this conceptual model, through alignment with the three axes, allows for a re-categorization of SADC TFCAs. Such a categorization recognizes the importance of agreements, whilst also recognizing their weaknesses, and allows for the recognition of additional considerations, such as alignment of national policy and legislation and the implementation and management efforts. This categorization would make provision for and recognize both top-down and bottom-up approaches in TFCA establishment. Top-down approaches, starts with high level ministerial buy-in and the drafting and signing of international agreements. Bottom-up approaches starts with the alignment and integration of management and implementation plans at operational level, with a view to then align national policy and legislation and, ultimately, culminates in the signing of international agreements. Both top-down and bottom-up approaches have different implications, and varied level of success in different contexts. However, in practice, there is also evidence of hybrid approaches to TFCA establishment that relies on parallel processes at international, national, and local levels. Ideally, all three categorizations should score highly if an ideal status is to be achieved. The above categorization is an attempt to present a more comprehensive approach to TFCA establishment, that recognizes progress beyond the mere signing of international agreements. A next step would be to apply this categorization to specific TFCAs, and thereby enhance our understanding of their status.

## Conclusion

This research makes explicit the weaknesses underpinning international TFCA agreements and thereby contributes to our understanding of TFCA development, progression and implementation. The current categorisation of TFCAs suggests that where formal agreements have been signed, TFCAs have been successfully established (Category A status). This is misleading because as this research shows, international agreements are particularly weak policy instruments that tell us little about the success of TFCAs. The three main weaknesses identified for the TFCA agreements included in this research are:

- *A lack of enforcement mechanisms (see Criterion 2, 3 and 8):* This weakness is not unique to TFCA agreements but to international environmental agreements generally (see for example Hafner-Burton et al. 2012; Iwata and Okada 2014; Koremenos 2016; Hoffman et al. 2022). Therefore, to address this mechanism, we need to look beyond the agreements themselves. The likely solution would be to rely on national level enforcement provisions, typically contained in national policy and legislation. Enforcement and the strengthening thereof in support of international agreements lies at the individual country level. TFCA categorization should, therefore, make provision to recognize such national level enforcement mechanisms.
- *Weak provision of resources for implementation (Criterion 4, 5 and 6):* Although commitment to the provision for resources can, and should, ideally be explicit in international agreements, implementation eventually happens at local level. This means that the lack of provision in the agreements themselves does not mean that no resources are provided. TFCA categorization would, therefore, do well to also recognize the extent to which resources are provided at a local level. *A lack of clear objectives (Criterion 10):* The TFCA objectives included in the international agreements are very high-level and vaguely defined. However, refinement of these objectives happens at local level management plans and implementation arrangements. The extent to which local level management and operational plans have been aligned with the agreements, and also across national boundaries, is critical for implementation. TFCA categorization should consider the extent to which these objectives have been operationalized in local aligned and/or integrated management plans.

The above weaknesses and limitations do not mean that the general concept of TFCAs is flawed, or that TFCAs are destined to fail. It suggests that the emphasis in the development of TFCAs should shift towards strengthening local conservation efforts (by aligning operational plans) and country specific governance and implementation (by aligning national legislation). Funding agencies, who is the life blood of TFCAs in the SADC context, could play a pivotal role by funding operational and legislative alignment in addition to securing agreements. Ultimately, our understanding of TFCA success (reflected in its categorization) must reflect a broader understanding and expectation beyond the formalization of international agreements. We trust that this research has made a meaningful contribution to achieve this.
